# A Positive Deviance-based Antenatal Nutrition Project Improves Birth-weight in Upper Egypt

**Published:** 2006-12

**Authors:** Mahshid Ahrari, Robert F. Houser, Siham Yassin, Mona Mogheez, Y. Hussaini, Patrick Crump, Gary L. Darmstadt, David Marsh, F. James Levinson

**Affiliations:** ^1^ Friedman School of Nutrition Science and Policy, Tufts University, Boston, MA 02111, USA; ^2^ Save the Children/Egypt Field Office, Garden City, Cairo, Egypt; ^3^ Department of International Health, Bloomberg School of Public Health, Johns Hopkins University, Baltimore, MD, USA; ^4^ Save the Children-USA, Westport, Washington, DC, USA

**Keywords:** Positive deviance, Pregnancy outcomes, Birth-weight, Pregnancy weight gain, First pregnancies, Antenatal care, Urinary tract infections, Daytime rest, Second-hand smoke, Behaviour change, Egypt

## Abstract

The positive deviance approach identifies and promotes existing uncommon healthy behaviours. A positive deviance-informed antenatal project was pilot-tested in Al-Minia Governorate, Upper Egypt, during 2003–2004, after a positive deviance study in 2000 found that successful pregnancies had increased consumption of meat and vegetables, daytime rest, and antenatal care; less second-hand smoke exposure; and symptoms of no urinary tract infection. Accordingly, health facilities were upgraded in target and comparison areas to provide quality antenatal care, including treatment of urinary tract infection. Additionally, in the target villages, women at-risk of delivering low-birth-weight infants were enrolled in weekly ‘IMPRESS’ (improved pregnancy through education and supplementation) sessions with counselling and supplemental food. In total, 519 women (344 target, 175 comparison) were enrolled in the third or fourth month of pregnancy and were followed through delivery. Birth-weights of the target mothers increased 2.2 times more than birth-weights of the comparison mothers over baseline (mean increase: 0.58 vs 0.26 g respectively, p<0.01). Similarly, the decrease in prevalence of low birth-weight from baseline was greater in the target villages than in the comparison mothers (% of decrease: 26.9 vs 11.9 respectively, p<0.01). The target at-risk women were far more likely than their counterparts to report eating more food (54.9% vs 10.6%), more meat (57.1% vs 4.2%), more vegetables (66.9% vs 5.3%), increasing daytime rest (64.1% vs 11.7%), and avoiding second-hand smoke (91.3% vs 51.6%) during pregnancy. The cost per 100 g of improvement in birth-weight was US$ 3.98. The Government of Egypt and partners are scaling up the elements of the project.

## INTRODUCTION

Pregnancy outcomes in Egypt are poorer compared to other countries of similar per-capita gross national product. While the national rate of low birth-weight (LBW) is 12%, almost one (30%) in three newborns weigh <3 kg at birth, and those weighing 2,500–2,999 g at birth have a 2.5-fold greater risk of death than those with birth-weight of 3,000–3,499 g (1–3). These increased risks of mortality associated with LBW continue through the first year of life and beyond (4, 5) and also increase the child's risk of cognitive disability, impaired emotional development, and poor school achievement (6). Results of recent research further indicate that surviving individuals with LBW are more likely as adults to have higher rates of death from coronary heart diseases and a higher prevalence of stroke, high blood pressure, and diabetes, each taking a toll on the productivity of these individuals and their communities, and increasing the burden on already over-stretched health systems (7).

The positive deviance (PD) projects have proven successful internationally in addressing a broad array of problems through identification of local solutions. Such problems have included illness and malnutrition among newborns, children, adolescents, and adults (8), inadequate exclusive breastfeeding (9), female genital cutting (10), and insufficient educational outcomes in the U.S. (11). PD is founded on the principle that, even in the presence of significant constraints, some individuals, households, or organizations have found ways to overcome these constraints to produce positive outcomes. Once these practices and their determinants have been identified, ideally with active participation of the communities involved, programmers can design and implement projects that increase the use of these positive practices—often using the positive-deviant individuals themselves as behaviour-change agents.

Save the Children-USA, in collaboration with the Maternal and Child Health Department of the Ministry of Health in Al-Minia Governorate, has been carrying out a PD-based strategy to deliver health and nutrition interventions to improve weight gain during pregnancy and birth-weight and to reduce maternal malnutrition and depletion in this part of Upper Egypt since 2000. This paper presents the results of the pilot phase of this project, which took place in 2003–2004.

## MATERIALS AND METHODS

### Positive deviance inquiry

During June-November 2000, the project partners carried out a PD inquiry in two large communities in Al-Minia, a traditionally less well-developed governorate. The PD inquiry was designed to identify those practices and characteristics associated with low-income women who managed to overcome economic and related constraints to have healthy pregnancy outcomes. Results of this PD inquiry, reported elsewhere (12), indicated that low-income women with good pregnancy outcomes were more likely to report multiple antenatal care visits; adequate daytime rest; increased consumption of meat, vegetables, oils, and dairy products; and an absence of symptoms suggestive of urinary tract infection (UTI) during pregnancy. In contrast, the practice of ‘eating down’ (that is, restricting the dietary intake to prevent a difficult delivery due to an overly large baby) during pregnancy was common among non-positive-deviant mothers. Analysis of these data also found that primiparous mothers were disproportionately represented among mothers delivering a LBW infant (55.5% vs 25.0% among non-primiparous women), while high-parity mothers were disproportionately represented among those with low gain in weight during pregnancy (mean gravidity 3.8 vs 3.0 among high-parity women).

### Project interventions and delivery strategies

Based on the results of this PD inquiry, we conducted social mobilization and training, and designed and launched a pilot PD project in March 2003 in two large target areas (Ghatousha and Deir-Samalout) with a single comparison area (Daghouf) in Al-Minia Governorate.* (This decision, made in close consultation with the Department of Health, accounts for the larger project than comparison population in this pilot project.) (*The procedures followed were in accordance with the ethical standards of the Internal Review Board of Tufts University.) The key strategies were upgrading antenatal care services and facilitating intensive weekly gatherings of pregnant women considered ‘at risk’ of delivering a LBW infant. The target areas received both the strategies, but the comparison areas received only the former.

#### Enrollment

In each project area, at the beginning of each month for six months, the project staff visited all pregnant women at home in the third or fourth month of pregnancy and invited them to a common weighing session for that cohort. All invited women accepted the proposal. With 180–200 women pregnant in a community at any point in time, each of these cohorts contained approximately 30 women. Each of the community health workers, trained by the project and responsible for managing and supervising anthropometric measurements, IMPRESS sessions, home-visits, and data collection in the target areas, supervised one cohort of pregnant women. To identify women considered at risk of delivering a LBW infant, nurses administered questionnaires using a pre-determined set of at-risk criteria. Women considered at risk were enrolled into the weekly IMPRESS (improved pregnancy through education and supplementation) sessions of food supplementation and counselling.

The project defined ‘at risk’ as: (a) women with low body mass index (BMI) (<22.6), a pre- or early pregnancy cut-off point for Egypt used by the World Health Organization on the basis of effects on pregnancy outcomes (13) at the first weighing, (b) women who failed to gain at least one kg in any month of pregnancy, and (c) all first pregnancies.

The project staff counselled the remaining pregnant women (not considered at risk) twice—once at their first weighing and once in the last month of pregnancy. Topics addressed included maternal nutrition and health, childcare, and infant feeding. Overall, in the two target areas, we enrolled 344 women, of whom 229 (66.6%) were enrolled in the IMPRESS sessions. Of the target women, 147 (42.7%) had a low BMI in early pregnancy, and 99 (28.8%) were first pregnancies.

In the comparison area, we enrolled 175 women, of whom 53 (30.3%) had low BMI in early pregnancy, and 67 (38.3%) were first pregnancies. The community health workers carried out enrollment in the comparison area.

#### IMPRESS sessions

We designed IMPRESS sessions to improve birth-weights in first pregnancies because birth-weights have tended to be lower in first births and because practices from successful first pregnancies seemed likely to be repeated in subsequent pregnancies. Trained volunteers, assisted by PD mothers and their mothers-in-law, counselled IMPRESS attendees on PD practices (12), plus promoted exclusive breastfeeding and timely and appropriate complementary feeding. Women continued to attend IMPRESS sessions until they delivered.

The IMPRESS sessions also provided a nutritious meal (consumed at the sessions) with selected foods identified through menus of PD mothers (12), including, per person: ¼ of a chicken or 150 g of red meat, ¾ cup cooked rice, one Baladi bread (local leavened bread weighing roughly 75 g, ½ cup cooked vegetables, one cup salad (tomatoes, cucumber, onion, and parsley) and fruit (totaling approximately 900 calories, 110 g carbohydrates, 55 g protein, and 30 g fat). Such meals motivated attendance, modelled a well-balanced menu, and provided calories and micronutrients.

The IMPRESS sessions maintained interest, enhanced learning, and built skills among participants through: (a) cooking-demonstrations and practice; (b) awards to IMPRESS attendees who had successfully put into practice the IMPRESS session messages (e.g. intake of iron/folate, intake of increased food, adequate daytime rest); (c) instruction on personal hygiene, including how to make and use home-made soap; and (d) assistance in preparing a birth-kit and selecting clothes and toys for newborns.

Mothers-in-law are often decision-makers in the homes of young married women. Accordingly, mothers-in-law of IMPRESS women were invited to join at least one pre-determined IMPRESS session, sessions which stressed the importance of antenatal care, diet, and rest and, specifically, the importance of helping pregnant women carry out daily chores. Husbands were also encouraged to visit the health clinic on pre-determined days to learn about the project and good antenatal care and to understand the importance of their wives avoiding second-hand smoke during pregnancy (a message now commonly accepted in antenatal care behavioural change).

#### Home-visits

The community health workers visited each IMPRESS attendee at home once a month to review messages, solve problems, and encourage practices not yet adopted. During enrollment in the IMPRESS session, the clinic nurses gathered information from attendees to identify those considered high risk according to particular criteria and in need of an extra home-visit every month.

High-risk factors included (a) first pregnancy, (b) absence of assistance in household chores, (c) extreme poverty, (d) more than three previous deliveries, (e) age of mother over 35 years, (f) a previous caesarian delivery, (g) a previous stillbirth, (h) previous neonatal death, (i) previous premature delivery, (j) maternal history of tuberculosis, heart diseases, or diabetes, (k) anaemia (haemoglobulin <11 g/dL), (l) hypertension (≥140/90 mmHg), (m) short stature (<150 cm), (n) history of disease needing referral to hospital, and (o) UTI.

The community health workers then tailored visits to the identified risk factors and counselled accordingly. If a participant missed a session, the home-visit conveyed the messages from that session.

#### Government antenatal care services

The Ministry of Health and Save the Children-USA, through the Maternal and Child Health Department partners, assured the availability of good-quality antenatal care for all women in both target and comparison areas, including iron/folate supplements, tetanus toxoid immunization, and diagnosis and treatment of UTI. Because of the known importance of UTI for pregnancy outcomes, again confirmed during the PD inquiry, we equipped and trained primary health centre staff to detect and treat UTI. All pregnant women in the target and comparison areas had frequent (usually monthly) antenatal care, where they were tested for UTI and were usually treated. Consistent with the Ministry of Health standards of care, providers charged women EL 1 or US$ 0.16 to treat each episode of UTI.

#### Socioeconomic status

Socioeconomic status was assessed in two ways during the project. At baseline, the community leaders were invited to group households into socioeconomic status categories of ‘very poor’, ‘poor’, ‘middle income’, and ‘rich’, based on land ownership, other assets, and income. This system, though adequate for the targeting of services within areas, was inadequate for comparison across areas. Accordingly, the International Food Policy Research Institute (IFPRI) was invited to adapt for use of the project an index of economic status it had developed for the Egyptian food subsidy programme based on the 1997 household expenditure data. The ‘proxy means test’, developed by Dr. Akhter Ahmed of IFPRI for use in this project, used regression models for predicting per-capita household-consumption expenditure using 14 explanatory variables and three statistically significant interaction terms. The variables included household size, the number of rooms in the house per person, the type of floor and walls of the house, total arable land, ownership of particular assets, number of livestock owned, business owned, and source of income. The household socioeconomic status scores were calculated by multiplying the household scores for each of these variables by coefficient for that variable emerging from regression analysis (In the case of variables with an interaction term, coefficient of the variable was added to coefficient of its interaction term.). The household scores in the project and control areas ranged from 42.38 to 6761.7, with a median score of 102.99.

#### Monitoring and evaluation

The clinic nurses measured birth-weights of all newborns within 48 hours of birth using a standard baby-weighing scale (Misaki digital baby scale [Misaki mb, Japan]) and obtained bi-monthly weight and length measurements. The Misaki digital baby scales, precise to 50 g, were calibrated prior to each weighing session by a project supervisor. Birth-weights were taken without clothing and were registered immediately in the appropriate project register. The quality-assessment team regularly reviewed birth-weight data collection and recording during their monthly visits.

To assure the quality of the project, Save the Children-USA and the Maternal and Child Heath Department selected a small team of experts knowledgeable about, but not involved in, project activities. The team—a nurse supervisor from the Ministry of Health Al-Minia Governorate, a gynaecologist from the local hospital, and a local laboratory technician trainer—made one field visit per month to observe and assess the quality of service provision and data collection. In addition, the team interviewed a sample of IMPRESS participants each month to assess their understanding of the messages. They reported to Save the Children-USA, and Maternal and Child Health Department immediately after each field visit to facilitate prompt response, if necessary. Overall, these reports indicated that the implementation of the project took place according to the project design and schedule. The field supervisors assessed the quality of anthropometric measurements for both women and infants, testing of UTI, and antenatal care more generally in both project and comparison areas. In project areas, this assessment also included food provided at IMPRESS sessions, group counselling, and home-visits.

The volunteers of the Community Development Association collected all data, except for IMPRESS high-risk assessment and anthropometric measurements which were obtained by the clinic nurse. We also carried out focus-group discussions with groups of women during pregnancy and after delivery.

Cost data were collected on an ongoing basis throughout the project. Cost data included costs of upgrading the Maternal and Child Health Department facilities, including laboratory facilities for the testing of UTI; costs of staff; staff training and staff transport; food provided at IMPRESS sessions; and monitoring and supervision costs.

### Data analysis

We conducted an evaluation of the project with pre- and post-implementation measures of pregnant women comparing those from target (IMPRESS, plus strengthening of the Ministry of Health services) vs comparison (strengthening of the Ministry of Health services only) communities. We measured birth-weights and reported pregnancy-related behaviours and use of health services. Some self-reported pregnancy-related behavioural data compared intake of food or rest during pregnancy with the practice prior to pregnancy. Use of data in analysis came from two cross-sectional studies carried out before and after the intervention occurred. Baseline birth-weight data were collected in the target and comparison areas over a three-month period, but, in each case, within 48 hours of the birth.

Sociodemographic variables included socioeconomic status, age, education and employment status of the mother, age at marriage, age at first pregnancy, number of pregnancies, number of liveborn children, and duration of time between last two pregnancies. Pregnancy-related practices included antenatal care services sought, intake of food, rest, and exposure to second-hand smoke during the previous month.

For continuous variables, the differences between villages were determined by parametric and non-parametric comparisons according to their degree of departure from normal distribution. Tests of statistical significance included Fisher's exact test, chi-square test, Pearson's correlation test, student's *t*-test, and ANOVA. All statistical tests were carried out using SPSS version 13.0 (14).

The analysis was stratified on the target/comparison areas and pre- and post-project status. Because of the baseline differences in the proportion of first pregnancies, we examined the effects of the project on birth-weights of infants delivered both from first and non-first pregnancies. We excluded twins from analysis. After cleaning of data and exclusion of cases with duplicate ID numbers and conflicting or inconsistent data, the total sample size was reduced from 614 to 519 subjects.

In the analysis, we defined ‘untreated cases of UTI’ as women who received a diagnosis of UTI but did not receive treatment. We defined first pregnancies as those not proceeded by a livebirth, realizing that our programmatic term differed from the more technical term, primigravida.

## RESULTS

### Characteristics of target and control areas

In the baseline survey, first pregnancies were more common among the comparison than among the target women (41.7 vs 25.9%, p<0.05) (Table 1). Accordingly, as indicated below, this variable was separately analyzed. The comparison areas also had a higher percentage of households characterized as ‘very poor’ or ‘poor’, although, in the subsequently-developed socioeconomic status index, the median scores in the target and comparison areas were virtually identical (102.48 vs 103.57).

**Table 1.** Characteristics of target and comparison areas at baseline

Characteristics	Comparison area (Daghouf) (n=48)	Target areas (Ghatousha and Deir-Samalout) (n=112)
No. of people	15,801	23,053
% of ‘very poor’ and ‘poor’	54.2 (26/48)	42.7 (59/112)
% of mothers with no schooling	83.3 (40/48)	74.1 (83/112)
First live/successful pregnancy for mother (%)	41.7 (20/48)	25.9* (29/112)
2 years or more spacing between youngest children among non-first pregnancies (%)	50.0 (14/28)	54.2 (45/83)
Age (years) of mother (mean±SD)	21.5±5.0	23.9±6.1

*Target and comparison difference significant at p<0.05;

SD=Standard deviation

The mean parity in the project and control areas was 3.45±2.42 and 3.18±4.0 respectively. Of women in the comparison area who met these same eligibility criteria, a higher proportion were first pregnancy than IMPRESS women (38.3 vs 28.8%), while a lower proportion had low BMIs in early pregnancy (30.3 vs 42.7%.) (Table 2). The median socioeconomic status score of women in the comparison area was nearly identical to that of the IMPRESS women (103.6 vs 104.8). The mean BMI in the project and control areas was 23.6±3.4 and 25.1±4.0 respectively.

**Table 2.** Pregnancy-related characteristics and practices of eligible pregnant women in target areas (IMPRESS participants) and comparison women (IMPRESS eligible in control area) at endline

Indicator	Comparison area (Daghouf) IMPRESS comparable (n=163)	Target areas (Ghatousha and Deir-Samalout) IMPRESS (n=229)
Food intake during pregnancy (%)		
More than usual	10.6 (10/94)	54.9 (101/184)*‡
Less than usual	34.0 (32/94)	8.7 (16/184)
Meat intake during pregnancy (%)		
More than usual	4.2 (4/94)	57.1 (105/184)*‡
Less than usual	12.8 (12/94)	7.0 (13/184)
Vegetable intake during pregnancy (%)		
More than usual	5.3 (5/94)	66.9 (123/ 184)*‡
Less than usual	11.7 (11/94)	9.2 (17/184)
Daytime rest during pregnancy (%)		
More than usual	11.7 (11/94)	64.1 (118/184)*‡
Less than usual	16.0 (15/94)	3.3 (6/184)
Exposure to second-hand smoke during pregnancy (%)	48.4 (45/93)	8.7 (16/184)*
Untreated cases of UTI (%)	35.6 (32/90)	13.6 (23/169)†
Intake of iron/folate pills (%)	80.6 (75/93)	97.5 (118/121)*
Intake of 7 or more iron/folate pills per week (%)	0 (0/75)	86.2 (100/116)*

*Target and comparison difference;

†Target and comparison difference significant at p=0.01; significant at p=0.01;

‡Significance test based on more vs not more between comparison and target areas

IMPRESS=Improved pregnancy through education and supplement;

SD=Standard deviation;

UTI=Urinary tract infection

### Behavioural change and use of health services

The target women increased their consumption of foods (including foods identified in the PD inquiry, i.e. meat and vegetables) and rest during pregnancy and had a greater intake of iron-folate and avoidance of second-hand smoke compared to their counterparts in the comparison communities with the same eligibility criteria (i.e. first pregnancy, low BMI in early pregnancy (<22.6) and/or inadequate weight gain during pregnancy [<1 kg per month)]) (Table 2).

### Birth-weight

The mean birth-weight was significantly higher at endline compared to baseline in both target and comparison groups (Table 3). However, the increase in birth-weight from endline to baseline was significantly higher (p<0.01) in the target areas (0.58 kg) compared to the comparison area (0.26 kg). The rate of LBW was significantly lower at endline compared to baseline in the target areas (2.6 vs 29.5%) than in the comparison area (6.9 vs 18.8%).

**Table 3.** Birth-weight and prevalence of LBW in target and comparison areas at baseline and endline

Study area	Baseline		Endline		
	Birth-weight (kg) mean±SD (n)	LBW % (no.)	Birth-weight (kg) mean±SD (n)	LBW % (no.)	Increased mean birth-weight from baseline (kg)
Target areas (Ghatousha and Deir-Samalout)	2.74±0.43 (112)	29.5% (33/112)	3.32±0.47* (344)	2.6%† (9/344)	0.58‡
Comparison area (Daghouf)	2.74±0.39 (48)	18.8% (9/48)	3.00±0.45 (175)	6.9% (12/175)	0.26

*Baseline and endline difference significant at p<0.01;

†Baseline and endline difference significant at p<0.01;

‡Change in birth-weight between the target and the comparison area significant at p<0.01

LBW=Low birth-weight;

SD=Standard deviation

The mean birth-weight for these at-risk women in the target areas was higher than for their counterparts in the comparison area (3.23 vs 3.01 kg) (Table 4). We found a yet more substantial difference in birth-weights between low socioeconomic status (<overall median socioeconomic status score of 102.99) households in the project and comparison areas (3.34 vs 2.99 kg) (Table 4). In the comparison area, with no targeted intervention to at-risk women, the mean BMI of women in early pregnancy who delivered high-birth-weight infants was 25.2, while the BMI of women who delivered lower-birth-weight infants (below 3,000 g) was 24.7, thus indicating that BMI, in this population, is not a good predictor of birth-weights.

**Table 4.** Endline birth-weights from IMPRESS participants vs eligible participants in comparison area and low SES group in target and comparison areas

Study area	IMPRESS/IMPRESS eligible birth-weight (kg)		Low SES (<overall median 102.99) birth-weight (kg)	
	No. of cases	Mean (±SD)	No. of cases	Mean (±SD)
Target areas (Ghatousha and Deir-Samalout)	229	3.23 (±0.47)**	344	3.34 (±0.45)**
Comparison area (Daghouf)	163	3.01 (±0.44)	175	2.99 (±0.44)

**Target and comparison difference significant at p<0.01

IMPRESS=Improved pregnancy through education and supplement;

SD=Standard deviation;

SES=Socioeconomic status

The project had less effect on first pregnancies vs non-first pregnancies (Table 5). Birth-weights in both the groups (first and non-first pregnancies) significantly increased between baseline and endline in the target and comparison groups, with the largest differences (0.66 kg) for non-first pregnancies in the target group. Additionally, the changes in birth-weight between baseline and endline for non-first pregnancies were significantly greater in the target than in the comparison group (p< 0.01).

**Table 5.** Birth-weight from first pregnancies and non-first pregnancies in target and comparison areas at baseline and endline

Study area	Pregnancy status	Baseline birth-weight (kg) mean±SD (n)	Endline birth-weight (kg) mean±SD (n)	Weight gain in pregnancy (kg)
Target areas (Ghatousha and Deir-Samalout	First pregnancies	2.75±0.44 (29)	3.13±0.51* (99)	0.38
	Non-first pregnancies	2.73±0.43 (83)	3.40±0.43*† (245)	0.67
Comparison area (Daghouf)	First pregnancies	2.71±0.43 (20)	2.95±0.53* (67)	0.24
	Non-first pregnancies	2.76±0.36 (28)	3.03±0.40* (107)	0.27

*Baseline and endline difference significant at p<0.01;

†Target and comparison difference significant at p<0.01;

SD=Standard deviation

Of the 344 infants delivered to project participants, 17 neonatal deaths occurred.

Correlation analysis showed that the intake of only iron/folate was significantly associated with birth-weight.

### Costs and cost-effectiveness

Table 6 presents cost and cost-effectiveness data for the project. As indicated, the IMPRESS intervention which involved not only the weekly counselling of at-risk pregnant women but also the provision of a meal was just over twice the cost of upgrading the health clinic. The total cost of the interventions per pregnancy was US$ 23.11, and the cost per 100-g gain in birth-weight was US$ 3.98.

**Table 6.** Annual project costs (pilot phase*) and projected annual project costs (scaling-up phase†)

Cost category and phase		Average gain in BW (g)	Cost per 300 pregnancies (EL)	Cost per pregnancy (EL)	Cost per 100-g gain in BW (EL)	Cost per 100-g gain in BW (US$)
Community organization and IMPRESS	Pilot phase	321	30,000	100	31.1	5.02
	Scaling-up phase	321	21,000	70.0	21.8	3.52
Health clinic upgrading	Pilot phase	260	13,000	43.3	16.7	2.69
	Scaling-up phase	260	13,000	43.3	16.7	2.69
Combined	Pilot phase	581	43,000	143.3	24.7	3.98
	Scaling-up phase	581	34,000	113.3	19.5	3.15

*Pilot phase cost in one village per year

†Based on project cost using reduced IMPRESS food costs

BW=Birth-weight;

IMPRESS=Improved pregnancy through education and supplement

## DISCUSSION

The project reduced the prevalence of LBW in this low-income area of Egypt. The prevalence of endline LBW in both target (2.6%) and comparison areas (6.9%) compared favourably with an all Egypt figure of 12% (15). The differences in reduction of birth-weight between the target areas and the comparison area were considerable in the study population as a whole among households of low socioeconomic status and among both first and non-first pregnancies. The results of ANOVA triple interaction (time*study area*pregnancy type) showed the highest mean change in birth-weight for non-first pregnancies in the target areas (p<0.01). The small number of first pregnancies in the target and comparison areas at baseline (n=29 and n=20 respectively) may account for non-significant effect among first pregnancies in the target women. However, the plot of means and 95% confidence intervals for first pregnancies and non-first pregnancies in the target areas and the comparison area at baseline and endline suggests that the intervention was also significantly effective for first pregnancies, although the ANOVA triple interaction was not significant. The greater effect of the project, particularly in the target areas, on non-first pregnancies—among whom the potential for higher birth-weights is biologically greater—suggests that this potential generally was achieved through the combination of improved healthcare and behaviour change. The project also increased significantly daytime rest, consumption of foods, intake of iron/folate, and avoidance of second-hand smoke among at-risk pregnant women in the project areas.

The dramatic effect of the project on birth-weights is somewhat surprising. The association between antenatal interventions (dietary supplementation and other interventions) and pregnancy outcomes is far less linear than the association between intake of food and child growth. Studies have found that the effects of pregnancy-related interventions on weight gain during pregnancy, birth-weight, child growth, and even birth-weight of the subsequent child depend on the degree and length of maternal deprivation. Other studies also have reported that the effects of pregnancy-related interventions were associated with birth-weight of the mother herself and of the mother's mother. The effects of pregnancy-related interventions on birth-weight are most pronounced in contexts of very low maternal energy intakes, specified in one study as <1,750 kcal/day (16). Rush reviewed the effects of pregnancy-related interventions noting that typical birth-weight increments were 20–50 g, except in cases of famine or seasonal deprivation where birth-weights increased by as much as 200 g (17). By contrast, the Egyptian women in this pilot project were relatively well-nourished, with only 2% having BMIs below 18.5, and few likely to be consuming less than 2,000 kcal/day.

Also puzzling is the observation that reported practices dramatically improved among the IMPRESS women, but correlation analysis failed to associate these changes with improvements in birth-weights. Several possible explanations might be advanced for absence of this association: (a) As in all survey research, there is no assurance that reported behaviour was, in fact, actual behaviour. (b) We designed the questionnaires to detect changes in food intake and daytime rest compared to pre-pregnancy intake or rest. This approach may have been inadequately sensitive to pick up the differences in intake or rest necessary to have an effect on birth-weight in this population with higher baseline birth-weights. (c) The research design did not measure factors known to benefit pregnancy outcomes, including the nurturing and special attention provided by the project staff, the mutual cooperation and support among women in the IMPRESS groups, and the support provided by family members who were directly counselled by the project and/or indirectly influenced by it.

The limitations of the study, therefore, include the inadequate quantification and sensitivity of data on practices and the exclusion of a substantial number of cases.

### The way ahead

The Ministry of Health and Population of the Government of Egypt, in cooperation with the Save the Children-USA and the Johns Hopkins Center for Communication Programs, has decided to scale up the project to cover additional districts in the Al-Minia Governorate and two additional governorates. Given the unusually high proportion of infants in the target areas found at baseline to be low birth-weight, the magnitude of improvement in birth-weight achieved in the target areas is unlikely to be replicated during the scale-up. As indicated in Table 3, the proportion of LBW infants in the target areas at baseline was nearly three times the national average, although the mean baseline birth-weights in the target and comparison areas were not statistically different. The project experience, however, has informed how and what to scale up. The improvement in birth-weights in the comparison area during the project period (Table 3) demonstrated clearly the effects of improved antenatal care alone on birth-weight. UTI, previously not widely appreciated as an important determinant of birth-weight in Egypt, is now understood to be an integral part of antenatal care services in Upper Egypt.

During the pilot project, all women with BMIs below 22.6, with failure to gain one kg per month, and with first pregnancies joined IMPRESS sessions. Based on the BMI results presented, we conclude that, unlike populations (e.g. Bangladesh) with a high proportion of very low BMIs (<18.5), BMI in Egypt is not a useful risk factor for low birth-weight. Accordingly, we recommend restricting eligibility for IMPRESS sessions to women with first pregnancies and to women with BMIs less than 22.6 who also fail to gain one kg per month. These refined criteria will limit enrollment in IMPRESS to about one-third of pregnant women, more feasible than the project experience in Deir-Samalout (54.0%) and Ghatousha (86.9%).

The project also demonstrated the added value of the IMPRESS strategy to bring at-risk women at the same stage of pregnancy together for counselling and active learning and to involve mothers-in-law. It is less clear how important the meals were to attract such women to the IMPRESS sessions. Accordingly, the Government of Egypt decided to reduce the expense of foods provided in these sessions during scale-up by limiting inputs to raw ingredients to be prepared by participants. Based on these reduced costs, but assuming the same project effectiveness, provides yet more attractive cost-effectiveness estimates for scale-up: US$ 3.15 per 100-g gain in birth-weight. The Figure presents the effects, on the mean birth-weight gain, of upgrading the health clinic alone and with IMPRESS activity, the latter both as carried out in this report (a prepared meal) and as planned in scale-up (provision of food ingredients). Substituting food ingredients for the prepared meals saves US$ 4.84 per pregnancy ($ 18.27 vs $ 23.11).

**Fig.**
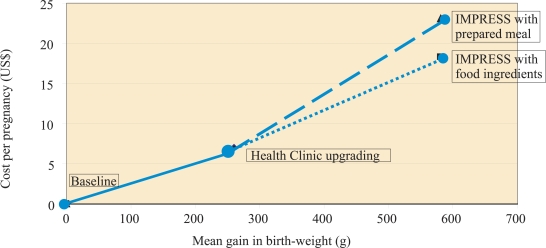
Cost-effectiveness of project activities

Finally, the project design team chose not to take on the challenge of over-weight and obesity even in this rural area of Egypt. Results of an initial analysis indicated that 24.5% of the pregnant women in the target and comparison areas had baseline BMIs of between 25 and 30 (the definition of ‘over-weight’), while 8.5% had BMIs over 30 (the definition of obesity). Together these women constitute one-third of the total women in the project. Data for Egypt as a whole are worrisome. Available data indicate that 70% of Egyptian women of reproductive age are now over-weight (higher than the U.S.), with nearly half of these defined as obese (18). And the trend is accelerating. Rates of over-weight are highest in urban areas. Even in rural areas, about half of women of reproductive age are over-weight.

Future iterations of this model could identify these women and provide them, perhaps also through weekly gatherings, with counselling and meals with less fat, fewer calories, and less processed carbohydrates.

## ACKNOWLEDGEMENTS

The authors acknowledge the contributions to this study made by the Maternal and Child Health Department, Ministry of Health and Population, Government of Egypt, Al-Minia. This research was supported, in part, by the Bill and Melinda Gates Foundation through a grant to the Saving Newborn Lives Initiative of Save the Children-USA.
